# Single-molecule kinetics of pore assembly by the membrane attack complex

**DOI:** 10.1038/s41467-019-10058-7

**Published:** 2019-05-06

**Authors:** Edward S. Parsons, George J. Stanley, Alice L. B. Pyne, Adrian W. Hodel, Adrian P. Nievergelt, Anaïs Menny, Alexander R. Yon, Ashlea Rowley, Ralf P. Richter, Georg E. Fantner, Doryen Bubeck, Bart W. Hoogenboom

**Affiliations:** 1 London Centre for Nanotechnology, University College London, London, WC1H 0AH UK; 2 Institute of Structural and Molecular Biology, University College London, London, WC1E 6BT UK; 3 Laboratory for Bio- and Nano-Instrumentation, Swiss Federal Institute of Technology Lausanne (EPFL), 1015 Lausanne, Switzerland; 4 Department of Life Sciences, Imperial College London, South Kensington Campus, London, SW7 2AZ UK; 5 School of Biomedical Sciences, Faculty of Biological Sciences, University of Leeds, Leeds, LS2 9JT UK; 6 School of Physics and Astronomy, Faculty of Mathematics and Physical Sciences, University of Leeds, Leeds, LS2 9JT UK; 7 Astbury Centre for Structural Molecular Biology, University of Leeds, Leeds, LS2 9JT UK; 8 Department of Physics and Astronomy, University College London, London, WC1E 6BT UK

**Keywords:** Nanoscale biophysics, Complement cascade, Atomic force microscopy, Reaction kinetics and dynamics, Biological physics

## Abstract

The membrane attack complex (MAC) is a hetero-oligomeric protein assembly that kills pathogens by perforating their cell envelopes. The MAC is formed by sequential assembly of soluble complement proteins C5b, C6, C7, C8 and C9, but little is known about the rate-limiting steps in this process. Here, we use rapid atomic force microscopy (AFM) imaging to show that MAC proteins oligomerize within the membrane, unlike structurally homologous bacterial pore-forming toxins. C5b-7 interacts with the lipid bilayer prior to recruiting C8. We discover that incorporation of the first C9 is the kinetic bottleneck of MAC formation, after which rapid C9 oligomerization completes the pore. This defines the kinetic basis for MAC assembly and provides insight into how human cells are protected from bystander damage by the cell surface receptor CD59, which is offered a maximum temporal window to halt the assembly at the point of C9 insertion.

## Abstract (web summary)

The membrane attack complex (MAC) is a hetero-oligomeric protein assembly that kills pathogens by perforating their cell envelopes. Here, the authors use atomic force microscopy to show that MAC proteins oligomerize within the membrane, allowing them to identify the kinetic bottleneck of MAC formation.

## Introduction

The formation of lethal membrane pores is a ubiquitous event in defence and attack between pathogens and their hosts^1–3^. It plays a critical role in the lytic, antimicrobial activity of human serum, the discovery of which was an early milestone in immunology^4^, and decades of study have unravelled the interplay between proteins that lead to the formation of immune pores in microbial membranes. The formation of these membrane attack complex (MAC) pores represents the final step in the activation of the complement system, an integral component of innate immunity, which surveys our body for pathogenic bacteria and which ‘complements’ the ability of leukocytes to kill pathogens. Dysregulation of MAC formation has been implicated in human disease^5,6^, and therapeutics that control complement are being harnessed for cancer immunotherapy^7,8^. Understanding how complement proteins assemble from innocuous soluble monomers into killer transmembrane pores can therefore contribute to developing strategies for treating human disease where the MAC is implicated^5^, and for repurposing the complement system as a potent immunotherapeutic^9^.

**Fig. 1:**
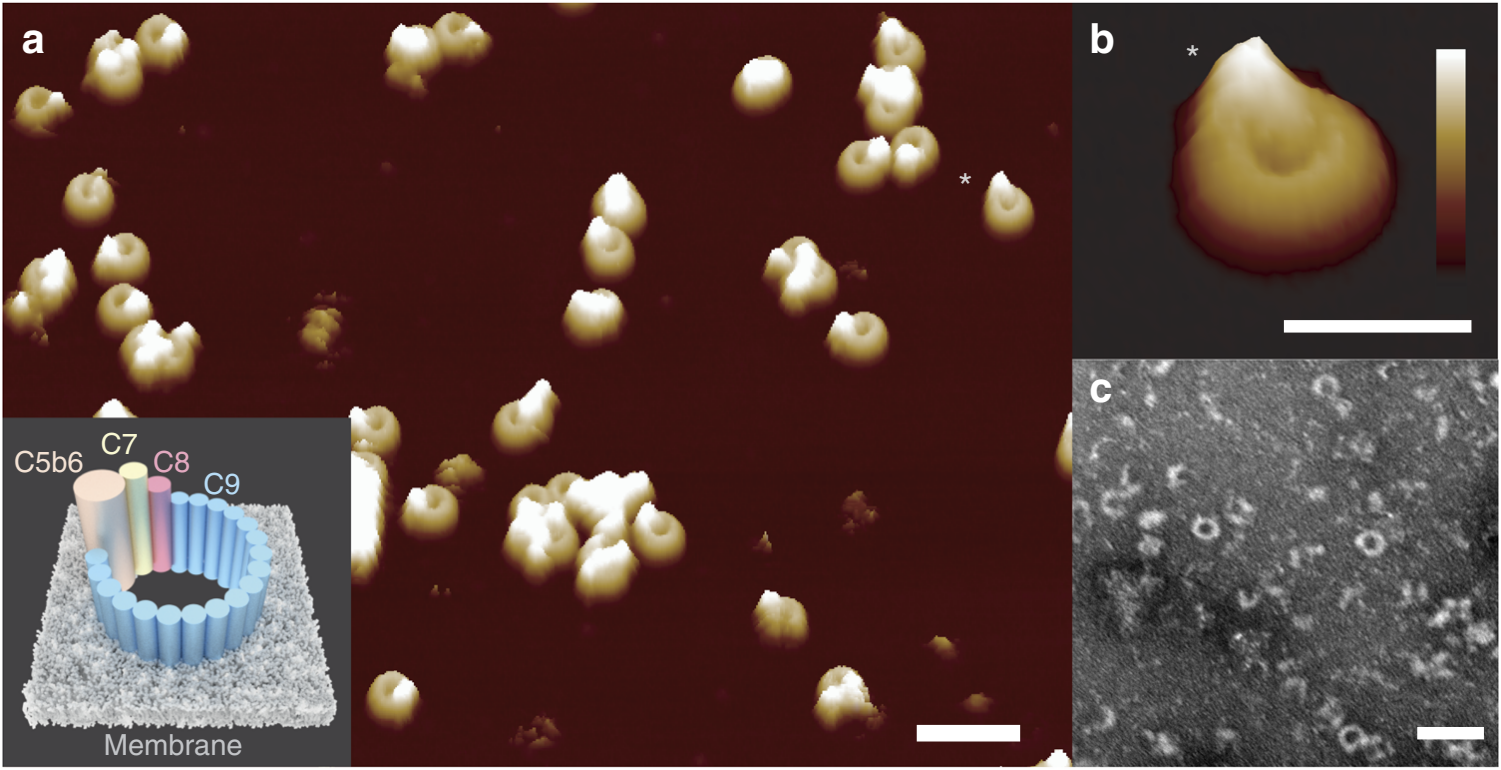
Formation of MAC on bacterial model membranes. **a** 3D AFM representation of the endpoint MAC pore structure on supported bilayers composed of an *E. coli* lipid extract. Inset: Schematic of the MAC, self-assembled from complement proteins C5b6, C7, C8 and C9, embedded within a lipid membrane. **b** Zoom-in of a single MAC pore (marked with an asterisk in **a**). **c** Negative-stain EM of *E. coli* lipid bilayers deposited on silicon dioxide grids, sequentially incubated with complement proteins C5b6, C7, C8 and C9, resulting in characteristic MAC rings observed in the bilayer membrane. Scale bars: **a**, **c** 50 nm, **b** 25 nm. Height scale (scale inset in **b**), **a**, **b** 20 nm

Assembly of the MAC is the end product of a complex series of biochemical interactions in which initially soluble complement proteins bind and undergo dramatic structural rearrangements to form a transmembrane pore. The resulting MAC pore is a hetero-oligomer formed from the irreversible, stepwise assembly of 7 different polypeptide chains: C5b, C6, C7, C8 (a hetero-trimer comprised of C8α, C8β and C8γ) and C9, where 18 copies of C9 are required to complete the pore (Fig. 1a, inset, Supplementary Fig. 1). Triggered upon detection of a pathogen, activation of complement leads to the generation of C5b via the cleavage of C5 by membrane-bound C5-convertase enzymes^10^. C5b is a metastable intermediate that rapidly sequesters C6^11^. Recruitment of C7 unfurls a lipophilic domain upon binding, while integration of C8 into the assembly is accompanied by an initial insertion into the membrane. The C5b-8 initiator complex then binds C9 and undergoes unidirectional, clockwise oligomerization (with 18 copies of C9) to complete an 11 nm wide transmembrane pore, as characterised in increasing structural detail by cryo-electron microscopy (cryoEM)^12–16^. Together with crystallographic structures of component proteins, high-resolution cryoEM analyses of the full pore have identified regulatory roles for auxiliary domains^15,17,18^ that control the transition from stable proteins in our blood to lethal transmembrane pores. However, it remains unclear which are the rate-limiting steps in the assembly pathway of these complement proteins.

As there is no known lipid or receptor specificity for MAC membrane insertion, the kinetics of pore assembly drives both the rapid innate immune response to pathogens and dictates how the MAC can be most effectively inhibited on membranes of self-cells. For human cells, the only known membrane-associated inhibitor of MAC assembly is CD59, a glycosylphosphatidylinositol (GPI) anchored cell surface receptor. CD59 binds the transmembrane residues of C8 and C9^19^, preventing pore formation and further oligomerization of C9^20^. The kinetics of MAC formation must allow a temporal window such that inhibitory factors can interfere at appropriate stages in the assembly pathway. Therefore, a kinetic analysis of MAC assembly will provide a much-needed framework to understand how CD59 inhibits lysis.

To understand the molecular mechanism and kinetics underpinning how and when the MAC assembly becomes cytolytic, we sought to track the progression of the complement terminal pathway at the level of single pores. Using rapid atomic force microscopy (AFM) imaging on supported model membranes, we visualize the initial interactions of complement proteins with the membrane, and resolve the kinetics of MAC pore formation. Together these data reveal the overall rate of the assembly process and identify which steps in the pathway are rate-limiting.

## Results

### MAC forms pores in bacterial model membranes

To enable AFM tracking of MAC self-assembly at single-molecule resolution, we developed a model membrane system that supported the formation of transmembrane pores. We sequentially incubated complement proteins C5b6, C7, C8 and C9 at physiological concentrations^10,21^ on supported bilayers formed from *E. coli* lipid extract^22^ and from *pseudo E. coli* lipid mixtures comprised of 1,2-dioleoyl-sn-glycero-3-phosphocholine (DOPC), 1,2-dioleoyl-sn-glycero-3-phospho-(1′-rac-glycerol) (DOPG) and 1,2-dioleoyl-sn-glycero-3-phosphoethanolamine (DOPE). In our model comprised of synthetic lipids, we sought to harness the physico-chemical properties of the bacterial membrane: PG lipids harbour negative charge in their phosphoglycerol headgroup, whilst PE introduces a degree of stored curvature elastic stress into the plane of the membrane^23^. Inspection by AFM and fluorescence recovery after photobleaching (FRAP) reveals a single continuous supported bilayer with rapid in-plane diffusion (Supplementary Fig. 2).

High-resolution AFM images of the resulting end-point MAC pores are consistent with cryo-EM reconstructions (Supplementary Fig. 3a–d). We clearly resolve the lumen of the β-barrel pore in addition to the protruding C5b stalk that hallmarks the MAC (Fig. 1, Supplementary Fig. 1)^12–16^. Vesicle lysis assays corroborate the functional requirement of both a C5b-8 ‘initiator’ and C9 ‘propagators’ for MAC to form lytic pores (Supplementary Fig. 3e); and negative-stain EM on lipid bilayers shows that C5b-8 is required for the oligomerization and insertion of C9 within the membrane (Supplementary Fig. 4). Together, these data confirm that the C5b-8 initiator complex, comprised of C5b6 in complex with C7 and C8, is essential for the formation of a functional MAC within the bacterial model membrane. Finally, we verified that an equivalent degree of MAC formation was observed across the lipid compositions used in this work (Supplementary Fig. 5).

### C5b-7 initiates MAC assembly on the target membrane

**Fig. 2:**
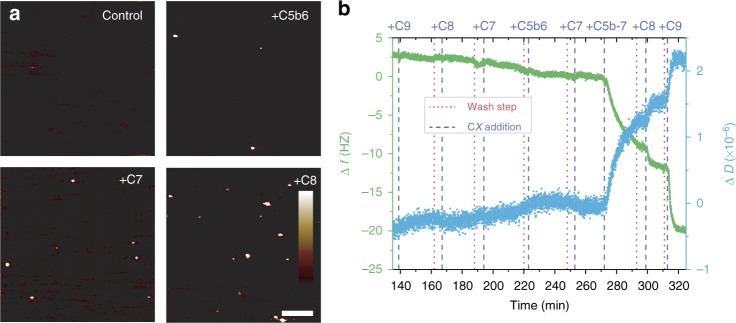
C5b-7 initiates MAC formation and recruits C8 and C9 directly from solution. Binding of complement components C5b6, C7 and C8 to supported bacterial model membranes containing PG lipids, as observed by AFM and QCM-D. **a** AFM images increasingly show protruding features upon addition of C5b6, C7 and C8 to a bilayer formed of DOPE:DOPG (50:50 mol%). Scale bar: 200 nm, height scale: 1 nm. **b** QCM-D binding assay following the addition of complement proteins, in reverse sequence followed by the forward sequence, to a lipid bilayer formed of DOPC:DOPE:DOPG (47.5:47.5:5 mol%) on a silicon dioxide coated QCM-D sensor. In isolation, complement proteins C7, C8 and C9 do not bind to the membrane, requiring C5b6 to initiate the interaction. Distinct steps in both frequency (Δ*f*) and dissipation (Δ*D*) upon sequential addition of C5b-7, C8 and C9 demonstrate protein binding to the membrane; wash steps (denoted by dotted red lines) between additions do not lead to a reduction in the signal size, revealing that the protein binding is stable. The dissipation shift increases upon binding, reflecting the relative softness of the protein complexes in the lipid bilayer film

Previous biochemical studies of MAC formation were performed on an ensemble of erythrocyte and liposome membranes, preventing analysis of individual pores^24–26^. By contrast, our experimental system facilitates a stepwise study of MAC assembly at the single-molecule level. Upon addition of C5b6 to a bacterial model membrane, we observe features of few-nanometre dimensions (Fig. 2a, Supplementary Fig. 6). Their transient nature on the membrane and protruding structure pose a challenge for AFM imaging^27,28^, in practice putting them at the detection limit of AFM. At higher sampling rates (Supplementary Movie 1), AFM force sensitivity was sacrificed for enhanced speed of imaging – in these instances, early assemblies are more difficult to detect. Further in situ incubation with C7 yields an increase in the number of such features, which appear with enhanced clarity after the addition of C8 (Fig. 2a, Supplementary Fig. 6). These data indicate that C5b6 interacts with the bacterial model membrane and becomes more resistant to the movement of the scanning AFM tip following assembly with C7 and C8.

To rule out artefacts due to the inherent invasiveness of the AFM measurement, we also performed binding assays by quartz crystal microbalance with dissipation monitoring (QCM-D) to study the protein assembly on/in membranes^29–32^, which correlate with our AFM data. Briefly, we prepared supported lipid bilayers on silicon dioxide coated QCM-D sensors, with which we could detect membrane binding of complement proteins^31,32^ (Fig. 2b, Supplementary Fig 7). Upon addition of C5b6, we observe shifts in frequency (Δ*f*) and dissipation (Δ*D*) with large variability between samples. Incubations with C5b-7 followed by C8 show consistently strong responses as stable complexes bind to the membrane and could not be removed by washing with excess buffer. From the analysis of the binding kinetics, and the C5b-7 dose-dependent C8 and C9 binding response, we show that the stoichiometry of the complex is determined by C5b-7 binding (Supplementary Fig. 8a). The QCM-D data also reveal that the protein complex becomes mechanically less compliant by its insertion into the membrane upon addition of C8 and C9 (Supplementary Fig. 8b)^33^. Upon addition of C9, we observe a final shift in frequency, corresponding to the inserted MAC pore. Taken together, these results demonstrate that the MAC is initiated by C5b6, which is explicitly required for C7 to bind to the membrane and propagate pore formation.

### C9 monomers are recruited directly from solution

MAC proteins are structurally homologous to the immune protein perforin and to bacterial cholesterol dependent cytolysins, both of which form pores through the homo-oligomerization of membrane-associated monomers^3,27–30,34–37^. We next explored whether the hetero-oligomeric MAC pore could assemble via analogous membrane-bound C9 oligomers, prior to its association with C5b-8. However, from the absence of C9 on *E. coli* lipid bilayers in the electron microscopy and AFM images in Supplementary Fig. 5, we conclude that such intermediates, if existing at all, are only transiently bound to the membrane. Upon addition of C9, AFM imaging (Supplementary Movie 1) does not show the characteristic pre-pore carpet that was detected by AFM for perforin^27^ and the cholesterol dependent cytolysin suilysin^28^. Instead, we observe a planar membrane background that remains featureless until MAC pores emerge, implying the absence of any pre-pore oligomers until the pore is assembled within the membrane. The static nature of these MACs confirms their membrane-inserted, pore character^27^. To further validate that C9 binds directly to C5b-8 and does not form C9 pre-pores on the membrane, we performed QCM-D analysis of bilayers that were incubated with complement proteins in the reverse order (i.e. C9, C8, C7, C5b6, Fig. 2b), with wash steps in between. C9, C8 and C7 separately did not show any significant binding to the membrane in the absence of other proteins. Taken together, these data demonstrate that C9 monomers are recruited directly from solution to the nascent pore and not via a membrane-bound C9 intermediate.

### Initial C9 insertion is a kinetic bottleneck in MAC assembly

**Fig. 3:**
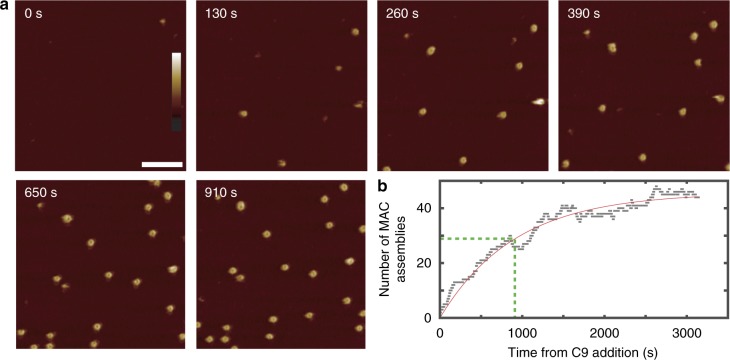
AFM shows that initial insertion of C9 is rate limiting in MAC assembly. **a** Time-lapse AFM imaging of MAC assembly on supported bilayers formed of *E. coli* lipid extract (see Supplementary Movie 1 for full data set). MAC assembly events initiate at distinct time points, with each MAC pore rapidly completing within a few frames; this implies a rate-limiting-step corresponding to initial insertion of C9. Scale bar: 200 nm; height scale (colour scale bar inset in image for *t* = 0 min): 40 nm. **b** Number of MAC assemblies detected (see Methods) with time, indicated as grey squares; the solid red line represents fitting with the function *A*(1–exp(–*t*/*τ*_init_)), where *τ*_init_ = 912 ± 32s represents the characteristic time of MAC pore appearance, highlighted by the dashed green lines. The completion time of each individual MAC is much shorter than *τ*_init_

By tracking the appearance and evolution of individual pores, we next determined the reaction kinetics that govern MAC assembly. Upon association with C5b-8, C9 oligomerizes to complete a transmembrane pore. With a frame rate of 6.5 s per frame, we used AFM to visualise C9 oligomerization in real time at 30 °C (Fig. 3a, Supplementary Movie 1, in which *t* = 0 is defined as the time of C9 addition); data recorded at the physiological 37 °C showed similar kinetics (Supplementary Fig. 9, Supplementary Movie 2). A single MAC pore is formed within the first few frames, immediately after addition of C9. The pore persists in isolation for 30 s, after which multiple (3–5) pores appear simultaneously, and the number of pores augments at a gradually decreasing rate, up to ~50 MAC pores in the field of view at the end of the recording (Supplementary Movie 1 and Fig. 3).

Remarkably, complete MAC pores continue to appear over an hour; yet the completion of each individual MAC occurs at the timescale of seconds to minutes (Fig. 3a, Supplementary Movie 1). Hence, two distinct kinetic steps are observed upon C9 addition to the nascent MAC. Firstly, we observe slow C9 binding to C5b-8, which is taken as our initial detection of a pore-forming event after the addition of C9. We define kinetics of this event by a characteristic initiation time *τ*_init_. Secondly, our data show rapid C9 oligomerization to a C5b-8C9_*n*_ MAC pore. We define the time for each growing pore to fully assemble as *τ*_olig_. Consistent with the description of these two distinct kinetic steps, the vast majority of end-point MACs in our data are complete, ring-shaped pores. If the rate of initial C9 insertion were faster than the subsequent oligomerization reaction, kinetically trapped, arc-shaped assemblies would occur due to monomer depletion, as has been observed for other pore-forming proteins^27,28,34^. To determine whether the kinetic bottleneck is attributed to C8 incorporation or insertion of the first C9, we allowed an extended temporal window after the addition of C8 and prior to adding C9: we confirm by time-lapse AFM imaging that this does not lead to more efficient/faster MAC formation (Supplementary Fig. 10, Supplementary Movie 3).

### Rapid AFM imaging allows quantification of reaction times

To quantify the reaction time of initiation and oligomerization, we analysed rapid AFM imaging data of pore assembly. Considering the up to 100-fold difference between the timescales of MAC appearance (initiation) and completion (C9 oligomerisation), the timescale of completion can be assumed negligible relative to that of initiation. We detected pores by cross-correlation during particle tracking (see methods), and take pore appearance as a proxy that reports on the time of initiation of MAC assembly after addition of C9 to the reservoir. In doing so, we determine a characteristic initiation time *τ*_init_ = 912 ± 32 s (Fig. 3b).

**Fig. 4:**
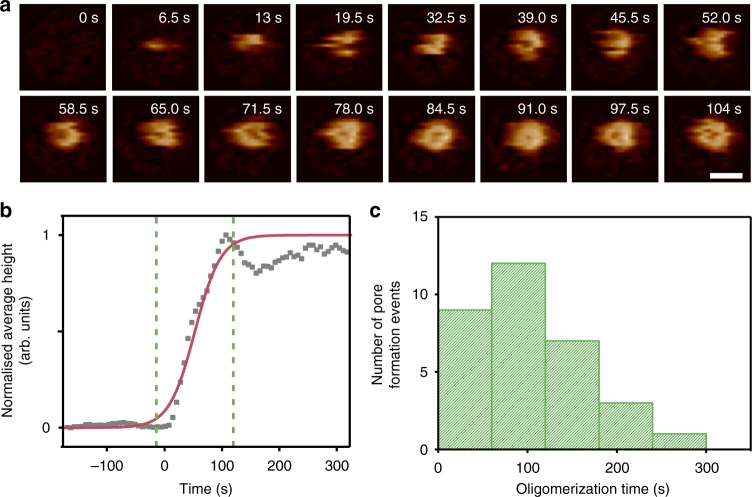
Real-time imaging of C9 oligomerization. **a** AFM image sequence of MAC assembly, cropped from data shown in Fig. 3 and Supplementary Movie 1. C9 oligomerization completes within frames shown (104 s, 6.5 s/frame; 0 s is here approximately defined by the frame preceding detection of the growing MAC). Scale bar: 30 nm, height scale (see colour scale bar in Fig. 1): 16 nm. **b** The normalised average frame height versus time for a single pore forming event (corresponding to event shown in **a**), plotted here as a measure for completion of MAC assembly. The red line represents a sigmoidal fit to the data, as a generic and mathematically convenient description of a smooth transition between pore absence and pore completion. The C9 oligomerization time is determined from the width of the transition, highlighted by green dashed lines (see Supplementary Fig. 12 for details). **c** Distribution of oligomerization times, extracted from *n* = 33 isolated pore forming events in 6 independent experiments

The kinetics of C9 oligomerization were determined by tracking areas with individual pores from just before the point of initial detection to after completion (Fig. 4, Supplementary Movie 4). Image sequences of these tracks show distinct intermediates of a growing pore as the MAC completes (Fig. 4a, Supplementary Fig. 11). To quantify the timescale of the transition we use the average frame height (defined by the average pixel intensity) as a proxy to report on the completeness of the pore. As the pore evolves we observe a gradual increase in the average height in the frame, which plateaus as the MAC reaches a final state (Fig. 4b, Supplementary Figs. 11–12, Supplementary Movie 4). Previous structural studies defined the stoichiometry of the complete MAC as having 18 copies of C9^12^. To measure the time required to add 17 copies of C9 after the initiation event, *τ*_olig_, we record the width of the transition between detection of the initial event and appearance of a complete MAC pore, *τ*_olig_ = 112 ± 17 s (Fig. 4c). This is an order of magnitude shorter than *τ*_init_, and implies that the average time per addition of each of the remaining 17 C9 subunits (*τ*_+_ = *τ*_olig_/17 = 6.6 ± 1.0 s) is more than two orders of magnitude shorter than *τ*_init_.

### A kinetic model for C9 assembly in the MAC

Given that $\tau _{{\mathrm{init}}} \gg \tau _ +$, we can interpret our data in terms of the separate reactions ${\mathrm{C5b}}{\hbox{-}}8 + {\mathrm{C}}9\mathop { \to }\limits^{k_{{\mathrm{init}}}} {\mathrm{C5b}}{\hbox{-}}8{\mathrm{(C9)}_1}$ and ${\mathrm{C5b}}{\hbox{-}}8{\mathrm{(C9)}}_n + {\mathrm{C}}9\mathop { \to }\limits^{k_ + } {\mathrm{C5b}}{\hbox{-}}8{\mathrm{(C9)}}_{n + 1}$ ($1 \le n \ < \ 18$), with respective rate constants *k*_init_ and *k*_+_. We observe that the C9 oligomerization times are independent of the time at which the reaction is initiated (Supplementary Fig. 13), consistent with C9 being in excess. As such, the interpretation of this reaction scheme can be further simplified by the assumption of an excess and thus approximately constant C9 concentration in solution ([C9] ≈ 1.4 µM) over the duration of our experiments. Consequently, under our experimental conditions the final number of MAC pores is set by the number of C5b-8 complexes on the membrane. These assumptions lead to the exponential time dependence of MAC appearance as observed in Fig. 3b, with *k*_init _≈ *τ*_init_^−1^[C9]^−1^ = 0.78 s^−1^ mM^−1^ (see Methods). In addition, assuming a constant rate of C9 addition after the insertion of the first C9, we find *k*_+ _≈ *τ*_+_^−1^[C9]^−1^ = 108 s^−1^ mM^−1^ (see Methods). This more quantitative analysis confirms that the initial insertion of C9, together with its binding to C5b-8, is the major rate-limiting step in MAC assembly. Interestingly, this rate-limiting step coincides with the stage where MAC pore formation is inhibited by CD59, which is present on the surface of human cells to prevent them from being lysed by complement^19,20,38^.

## Discussion

The MAC represents a biomedically important system in which to probe how unique individual proteins self-assemble into a macromolecular functional unit. Once initiated, five soluble complement proteins sequentially and irreversibly self-assemble into a hetero-oligomeric pore that opens up an 11 nm hole in a fluid lipid bilayer. While there is an extensive (and still expanding) body of structural and functional information documenting the MAC and its constituent components, the pathway and kinetics of its assembly have been more difficult to study^24^. Here we have presented rapid AFM imaging data that track MAC assembly at the single-pore level in real-time. Furthermore, we have derived kinetic models for initiation and oligomerization of C9 that explain how rate-limiting assembly intermediates can be captured by our body’s self-defence mechanism to prevent disease.

**Fig. 5:**
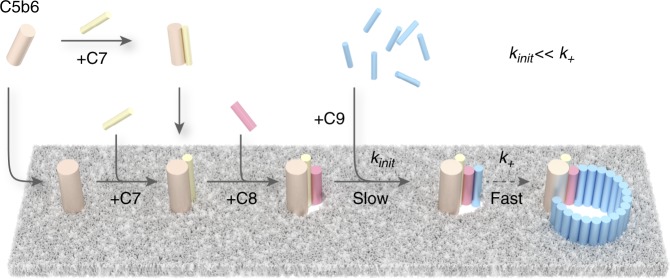
Schematic of MAC assembly. Upon formation, C5b6 templates the assembly, recruiting C7 and C8 to the nascent MAC. The C5b-7 complex is irreversibly bound to the membrane. Insertion of the first C9 is the rate limiting step in the MAC assembly; once this barrier has been overcome, subsequent C9 binding/insertion occurs more than two orders of magnitude faster than the initial C9 insertion

Based on the results presented here, we define a kinetic pathway of MAC assembly (Fig. 5). C5b6/C5b-7 binds to bacterial lipids and serves as a platform for coordinating the sequential assembly of the MAC at the target membrane, recruiting C8. Downstream, the C5b-8 initiator complex is explicitly required for membrane insertion of the initial C9 molecules. Our data reveal that this initiation phase, which relates to the insertion of the first C9, is the rate-limiting step. A rapid oligomerization phase completes the transmembrane MAC pore, as further copies of C9 bind and insert into the membrane directly from solution. Specifically, the binding of the initial C9 to C5b-8 is characterised by a rate constant that is more than two orders of magnitude smaller than that for the subsequent binding of C9 to C5b-8C9_n_ (1 ≤ *n* < 18), and occurs more slowly than C5b-8 formation.

Since the propagation of C9 oligomerization is so much faster than the initiation, we predict that there will be a low probability of incomplete, arc-shaped assemblies that are kinetically trapped due to monomer depletion. In agreement with this prediction, we find that MAC assembly favours formation of complete, ring-shaped pores (Fig. 1a), in contrast to kinetically trapped arc pores observed for other pore-forming proteins^27,28,34^. In summary, we show that once activated, the MAC pore rapidly assembles into complete ring lesions in the target membrane.

By having distinct initiation and propagation stages, C9 assembly in the MAC resembles pore formation of the related immune protein perforin, where the rate limiting step is the insertion of a small (membrane-bound but not yet membrane-inserted) pre-pore assembly, acting as a nucleation site from which to grow a transmembrane pore^27,39^. Although we do not observe any membrane-bound C9 pre-pore assemblies, the C5b-8 initiator complex, together with a C9 molecule yet to undergo its transmembrane transition, could serve a similar function. Our data provides evidence that MAC is a growing pore, similar to perforin. Distinct initiation and propagation stages have also been proposed for bacterial cholesterol-dependent cytolysins (CDCs), with the formation of a stable membrane-bound dimer followed by the addition of further monomers^35^. However, the CDCs differ from the MAC and perforin in that they undergo a concerted oligomeric pre-pore-to-pore transition, after which no further assembly events have been observed^3,28,34^. In a bacterial membrane that harbours a dense proteinaceous network of porins^10,40^, such a collective pre-pore-to-pore transition is likely to face a large free energy barrier; instead, a growing pore that directly recruits individual monomers from solution can act as a jack that prises open a hole within the porin lattice.

The transition from soluble monomers to a transmembrane β-barrel pore requires dramatic conformational changes in the pore-forming domain of complement proteins. The membrane attack complex-perforin/cholesterol dependent cytolysin (MACPF/CDC) domain is comprised of a central kinked β-sheet with two helical sub-regions (TMH; transmembrane hairpins) that unfurl to form membrane-inserted β-hairpins. This transition is accompanied by unbending of the central β-sheet and the displacement of a latch that releases the TMH bundles. For CDCs, this latch is manifested as a fifth β-strand of the central sheet that upon pore-formation converts to a helix-turn-helix, enabling oligomerization^36,37^. For MACPF-containing complement proteins, the latch remains helical and moves as a unit to release transmembrane residues^13,15^. Interestingly, the rate-limiting step of MAC assembly, as identified here, coincides with unfurling of the hairpins of the first C9 into the membrane. This can be related to the recent structural insight that C9 can bind to the C5b-8 initiator complex before inserting into the membrane; however, to propagate oligomerization, it requires conformational changes with the MACPF domain that accompany membrane insertion^15^.

Our results show that C5b-7 initiates MAC assembly at the membrane with subsequent protein components C8 and C9 integrated directly from solution. Upstream, the C5b6 crystal structure demonstrated that prior to interacting with membrane lipids, C6 membrane-interacting residues remain in their soluble helical form^17^; however, the C5b6 complex has been shown to associate with lipid bilayers^13^. The dual behaviour of C5b6 is reflected in the variability in our results (Fig. 2b, Supplementary Fig. 7), suggesting that interactions are mediated by a combination membrane defects and electrostatics^13^. It is conceivable that C5b6 membrane-binding is dominated by electrostatic interactions of negatively charged lipid headgroups (such as PG lipids commonly found in Gram-negative bacteria^41^) with either the thrombospondin (TS)1 domain of C6^42^ or by its unfurled membrane interacting β-hairpin^13^, both of which expose an interface rich in positive charge. Such lipid headgroup dependence also emerges from studies on MAC binding^31,32^ and complement activation on model membranes^43^. The observed membrane binding of C5b6 is consistent with recent work on bacteria^10^, reporting that the downstream efficiency of MAC formation is greatly enhanced when C5b6 is actively formed by C5 convertases bound to the bacterial surface. Our results here provide the rationale for this observation. We propose that nascent C5b6 can, under certain conditions, interact with the bacterial membrane upon its generation by the C5 convertase. C7 greatly enhances the membrane-bound stability of the nascent complex as it unfurls a lipophilic domain, and recruits C8 directly to the target membrane. This facilitates binding and oligomerization of C9, thus generating the functional pores that kill the bacterium.

Although the here discussed lipid dependence suggest some MAC specificity for bacterial targets, C5b-8-initiator complexes can deposit and progress to cytolytic pores on host cells if not properly controlled. Therefore, human cells express CD59 on their surface, which disrupt MAC assembly from the point of C5b-8 formation onwards^19,20,38,44,45^. Specifically, CD59 interacts with MAC precursors at two distinct stages. Firstly, CD59 can bind after incorporation of C8 (C5b-8) and prevent bilayer perforation by the C8α chain^44^. Secondly, CD59 can bind after addition of C9 (C5b-9) to inhibit further oligomerization and pore formation^44^. CD59 inhibits MAC assembly by binding to the TMH β-hairpin on the leading-face of C8α (residues 334–385)^45^ and to a buried 6 amino acid sequence of C9 (residues 366–371) that is exposed upon binding C5b-8^38^. However, the structural basis for how CD59 inhibits complement proteins is unclear. It is not known whether C9 initiates insertion of its hairpin into the membrane prior to binding CD59 or if CD59 binds C8 and C9 simultaneously.

Our data highlight a rate-limiting step at the association of C9 with the C5b-8 MAC precursor. This allows a maximum temporal window for the mechanism by which human cells are protected from autoimmune attack by the MAC: our model favours a mechanism whereby C9 has not unfurled into the membrane immediately upon binding C5b-8, allowing CD59 to inhibit MAC formation. The here reported initiation step would thus correspond to the insertion of the first C9 into the membrane, facilitating fast recruitment of additional C9 to the growing transmembrane complex. When CD59-mediated inhibition is overcome by antibody-based drugs such as rituximab that facilitate MAC-induced killing of chronic lymphocytic leukaemia B-cells, cell death follows at the ~100 s time scale^7^. This is consistent with the oligomerization kinetics of a single pore observed here in vitro, suggesting that only few pores are sufficient to lyse a B-cell.

In summary, we have determined the pathways and kinetics of assembly for a hetero-oligomeric protein complex by molecular-scale measurements. These assembly kinetics govern how MAC kills bacteria and how our body’s self-defence mechanism prevents membrane damage, which may also be relevant for complement dependent cytotoxicity in cancer immunotherapy^7^. Finally, we anticipate that our findings will guide the interpretation of ongoing studies towards an atomistic model of MAC structure.

## Methods

### Materials

*E. coli* lipid extract (total), 1,2-dioleoyl-sn-glycero-3-phosphocholine (DOPC), 1,2-dioleoyl-sn-glycero-3-phosphoethanolamine (DOPE), 1,2-dioleoyl-sn-glycero-3-phospho-(1′-rac-glycerol) (DOPG) and 1-palmitoyl-2-(dipyrrometheneboron difluoride)undecanoyl-sn-glycero-3-phosphocholine (TopFluor® PC) were purchased from Avanti Polar Lipids (Alabama, USA) as a powder and stored at −20 °C prior to use. Complement proteins C5b6, C7, C8 and C9 were purchased as proteins purified from human serum from CompTech (Texas, USA) and stored at −80 °C prior to use. For the rapid AFM imaging experiments, lysis assays, negative-stain EM and FRAP experiments shown here, *E.coli* lipid extract was used. For AFM imaging of intermediates, an equimolar mixture of DOPG:DOPE was used. For QCM-D binding assays, a lipid mixture of DOPC:DOPE:DOPG (47.5:47.5:5 mol%) was used, as formation of continuous bilayers containing high molar ratios of charged lipid ( >30 mol%) were not attainable on the silicon dioxide QCM-D sensors used here^46^.

### Preparation of lipid vesicles

Pure lipids were dissolved in chloroform at 10 mg/mL and mixed in solution to give a lipid mixture at a desired molar ratio. The lipid-in-chloroform solution was then dried in a glass vial under a stream of nitrogen gas to give 1 mg of lipid as a thin film. The lipid film was hydrated in buffer (20 mM HEPES, 120 mM NaCl, pH 7.4), vortexed and bath sonicated to give a cloudy lipid suspension. The suspension was then passed through a 50 nm polycarbonate membrane (GE Healthcare Lifesciences) 15 times to yield a clear suspension of small unilamellar vesicles (SUVs). All lipid species used had a gel-to-fluid transition below room temperature, and therefore were assumed to be miscible without heating.

### AFM sample preparation

Supported lipid bilayers were formed by injecting 4.5 µL of the SUV suspension to a freshly cleaved mica disk (6 mm diameter) under 18 µL of incubation buffer (20 mM HEPES, 120 mM NaCl, pH 7.4). CaCl_2_ solution (2.5 µL, 100 mM in incubation buffer) was added to give a final calcium concentration of 10 mM; this induces the rupture of the vesicles onto the mica support over an incubation period of approximately 30 min. Excess vesicles were then removed from the supernatant by rinsing with 500 µL of incubation buffer, to yield a uniform bilayer free of adsorbed vesicles (as assessed by AFM imaging). All SUVs were incubated at room temperature, above their gel-to-fluid transition temperature.

Endpoint MAC pores were formed by incubating the supported bilayer in a humid chamber at 37 °C and sequentially adding complement proteins C5b6, C7, C8 and C9 at 5 min intervals, with a final 15 min incubation after the addition of C9 prior to initiating the AFM experiment. Final concentrations of complement proteins were ~80 nM for C5b6, C7 and C8 and 1.4 µM for C9. Excess soluble protein was removed prior to imaging by washing with 5× sample volume (25–50 µL) of buffer.

‘Real-time’ MAC samples were formed in situ within the AFM liquid chamber. For experiments at 37 °C using a Bruker Dimension FastScan, the complement proteins were injected directly onto the supported lipid bilayer whilst imaging. For rapid imaging experiments performed at 30 °C using home-built AFM instrumentation described previously^47^, the sample chamber was passivated with BSA (0.1 mg/mL in incubation buffer) for 15 min and rinsed with 500 µL buffer prior to loading the sample with the supported lipid bilayer. Complement proteins were sequentially injected though channels in the sample chamber to the imaging volume, while scanning and in the absence of wash steps, to give a final protein concentration of 80 nM for C5b6, C7 and C8 and 1.4 µM for C9.

### AFM Imaging

AFM imaging was performed in fluid using a Bruker Dimension FastScan (for time-lapse imaging at 37 °C), a Bruker BioScope Resolve (for imaging of MAC intermediates) and a home-built instrument with rapid imaging capabilities^47,48^ (for rapid imaging at 30 °C). Imaging was generally performed in off-resonance tapping/fast force-feedback imaging (Bruker’s PeakForce Tapping) mode where force-distance curves were recorded at either 8 or 32 kHz, with amplitudes of 10–20 nm. With these frequencies, images could be collected at 5–100 s/frame. Tapping mode of MAC intermediates was performed while scanning bidirectionally (i.e. turn around at the end of each imaging line; no ‘retrace’).

Rapid PeakForce Tapping (with the z scanner driving at 32 kHz) was performed with largely custom built hardware as described in detail elsewhere^47^. Importantly, the AFM head has a sufficiently small laser spot to accommodate miniaturised cantilevers, and the high-speed scanner is flexure-based with a 1.8 µm × 1.8 µm × 2 µm range and ~100 kHz *z* bandwidth. Fast force–distance based imaging modes were implemented by sinusoidally modulating the tip-sample distance at a high rate between 16 and 32 kHz and recording the resulting deflection signal with a significantly higher sampling rate (512 kHz). The resulting periodic hydrodynamic background was recorded slightly above the surface and subtracted from the deflection in real time. The resulting interaction was a sinusoidal force-distance curve where the maximum force was used for feedback. For practical purposes, highest-quality data were recorded at 30 °C; and next compared with results obtained at the physiological 37 °C (see main text).

Commercial FastScan-D cantilevers (Bruker) were used for all experiments, except for images shown in Fig. 2, which used pre-release Fast Tapping probes (Bruker; resonance frequency 140 kHz, spring constant 0.3 N m^−1^). FastScan-D cantilevers have a specified spring constant of 0.25 N m^−1^ with a resonance frequency of 110 kHz in liquid; this exceeds our ramping frequency by at least a factor of 3, sufficient to avoid coupling between the ramping frequency and the cantilever resonance. Cantilevers were rinsed in isopropanol:ethanol (1:1) and plasma cleaned in air prior to use.

### AFM data processing

Image analysis was performed using Nanoscope Analysis version 1.80 (Bruker)^27^. Briefly, images were plane levelled and line-by-line flattened with the lipid bilayer as a reference. A Gaussian filter with a full-width half-maximum of 2 pixels (corresponding to 4 nm) was used to smooth out high frequency noise where necessary.

Tracking the evolution of a growing pore, and pore counting, was performed as follows, using MATLAB (MathWorks), and the scripts described were used with Bruker’s MATLAB toolbox: NSMatlabUtilites.

AFM Movie sequences were loaded into MATLAB, and a 1st order plane background subtraction was applied to each image. A template pore was user-selected from the final image in the sequence. This was used as the template in a 2D cross-correlation analysis, applied to each image in the sequence. If features correlated with the template over a given, normalized threshold value (set as 0.6 here; user-adjusted to optimize recognition), such features were identified as MAC pores. The number of features found in each frame was defined as the pore count. Next, using the coordinates already obtained from the 2D cross-correlation analysis, the coordinates of the appearance and growth of unique pores were tracked, and their coordinates stored into a new array (track). This used two further parameters: (i) A maximum linking distance (typically set at ~30 pixels), which defines a pore as being the same unique pore as that detected in the previous frame, only if the coordinates of the feature were within the maximum linking distance. (ii) A maximum gap closing (in frames), which defines the number of frames in which a feature (that is within the maximum linking distance) cannot be found and yet is still defined as belonging to the same track (this reduces the risk of artefacts due to image noise). The pore count and coordinates for individual tracks were then saved into a data structure.

Next, using the track coordinates, new image sequences for each pore were cropped to within a radial distance of 25 nm from the centre of the feature, and including some extra frames recorded before the first appearance of a given pore. For each cropped image sequence, the average height of each frame in the sequence was calculated and saved into a new array. This analysis was repeated for several data sets, and the data saved into new data structures. Finally, the cropped image sequences and average height arrays from several experiments were concatenated. Each track was inspected by the user and any false positives (caused by having too small a threshold value for the 2D cross-correlation analysis) were removed. A Savitzky-Golay filter was applied to each remaining average height array to reduce the effect of image noise. A sigmoid function of the form

1 $$f\left( t \right) = A \times tanh\left( {\frac{{\left( {t - t_0} \right)}}{\tau }} \right) + B$$

was fitted to each filtered, average height array. *A*, *B*, *t*_0_, and *τ* are fitting parameters, and 3*τ (which corresponds to ~90% of the transition; see Supplementary Fig. 12) was defined as the width of the transition (and hence reaction time). If, from the fitting, *t*_0_ was negative or 3*τ was longer than the Movie sequence, this was considered a poor fit and the data was removed. For the image sequence tracking a single MAC pore in Fig. 4, a 1.5 nm Gaussian filter was applied.

### Pore cross-sectional measurements

Image processing was carried out in Gwyddion^49^. Images were plane levelled and line-by-line flattened with the lipid bilayer as a reference. A Gaussian filter with a full-width half-maximum of 2 pixels was used to smooth out high frequency noise. Following this, a cross-section was then taken diagonally across a single MAC pore (shown in Fig. 1.) and exported. Data was plotted in Origin.

A MAC pore of structure EM-3134^12^ was imported into Chimera^50^. Volume filtering was performed with *σ* = 14 pixels to obtain a structure with resolution comparable to that of the AFM data. Given a resolution of 8.5 Å for EM-3134, this corresponds to a physical width (of the filter) of 11.9 nm. The volume viewer tool was optimized to ensure the full surface as shown (level 0.002). The model was then coloured by height with a black to white gradient (low to high) of −100 to +200 Å in steps of 75 Å. A top view image was then exported into Gwyddion^49^ for cross-sectional analysis. A cross-sectional measurement was taken horizontally across the pore diameter against the approximate height of a surrounding membrane (50 Å), including the stalk, and exported. Data was plotted in Origin.

### QCM-D measurements

Quartz crystal microbalance with dissipation monitoring (QCM-D) allows semi-quantification of mass deposition to a quartz crystal sensor. Binding assays were performed by flowing complement proteins over a lipid bilayer supported by the silicon oxide coated QCM-D sensor. Biomolecules interacting with the sensor interface give rise to a change in resonance frequency (Δ*f*) and energy dissipation (Δ*D*) of the quartz sensor. Briefly, a decrease in resonance frequency is proportional to an increase in surface-bound mass (where this includes hydrodynamically coupled solvent in addition to the biomolecules binding), whilst an increase in dissipation qualitatively correlates with an increase in the ‘softness’ of the film^33^. QCM-D measurements were performed in flow mode at a flow rate of 10 μL/min using a Q-Sense E4 system equipped with four Q-Sense Flow Modules (Biolin Scientific, Vastra Frolunda, Sweden) with a working temperature of 20 °C. Silicon oxide coated QCM-D sensors (QSX 303, Biolin Scientific) were used as substrates for supported lipid bilayers. Before injection, C5b6, C7, C8 and C9 were diluted to concentrations of 10, 5, 5 and 50 μg/mL (35, 54, 33 and 704 nM) respectively in incubation buffer (roughly equivalent to half those use in AFM experiments). Overtones *j* = 3, 5, 7, 9, 11, and 13 were recorded in addition to the fundamental resonance frequency (4.95 MHz). Changes in dissipation (ΔD) and normalised frequency, Δ*f* = Δ*f*_*j*_*/j*, for *j* = 5 are presented here; all other overtones provided equivalent information.

### Vesicle lysis assays

*E. coli* lipid extract was suspended at 7.5 mg/mL in calcein solution (50 mM calcein, 150 mM NaCl, 20 mM Hepes pH 7.4), freeze-thawed 6 times (liquid nitrogen – 65 °C) and extruded through a 100 nm polycarbonate membrane (Whatman) to form unilamellar calcein-encapsulated liposomes. Non-encapsulated calcein was removed through liposomes purification on a gravity-flow Sephadex-G50 (GE Healthcare) column (500 mM Sucrose, 150 mM NaCl, 20 mM Hepes pH 7.4) and liposomes were used immediately. MAC lysis assays of liposomes were performed by sequential addition of C5b6 (5 min, 37 °C), C7 (5 min, 37 °C), C8 and C9 at a mass ratio of 1:1:1:1. In control conditions, identical volumes of protein buffer (120 mM NaCl, 10 mM Hepes pH 7.4) were added instead of protein. Self-quenched encapsulated calcein was un-quenched through its release in the extra-liposomal solution following MAC lesions. Fluorescence was recorded immediately following C9 addition and every minute for 60 min on a SpectraMax M2 fluorometer (dual monochromator, ex: 490 nm, em: 520 nm) (Molecular Devices). Background fluorescence was measured from calcein-encapsulated liposomes in the absence of protein and subtracted from the data. To determine the percentage of lysis, the fluorescence was then normalized to the maximal lysis fluorescence estimated after a freeze-thaw cycle of liposomes incubated in 0.25% sodium dodecyl sulphate (SDS). Fluorescence measures of lysis and controls were always performed on the same batch of liposomes and in three independent replicates.

### Negative-stain EM

Supported lipid bilayers were formed as described for equivalent supported lipid bilayers as used in AFM experiments, using 8 nm thick PELCO® silicon dioxide support films for transmission electron microscopy grids (Agar) as the support instead of mica. Briefly, the glow-discharged grids were incubated with an SUV suspension in calcium containing incubation buffer (20 mM HEPES, 120 mM NaCl, 10 mM CaCl_2_), rinsed and incubated with complement proteins as described above. Importantly, this allowed us to remove all soluble protein and excess lipid from incubation buffer prior to staining. Samples grids were rinsed with 500 µl incubation buffer, taking care that they remained hydrated throughout, and subsequently stained with 2%wt/wt uranyl acetate. The sample was incubated with uranyl acetate for 60 s and carefully blotted dry, ensuring that the strain was quickly removed to avoid crystallisation of excess uranyl acetate at the surface. Samples were imaged on a Tecnai T12 thermionic filament microscope (Thermo Fisher Scientific) at 120 kV. Images were taken with a defocus of 0.5–1 µm on a Gatan 4 k × 4 k CCD camera, giving a final pixel size of 1.64 Å.

### Fluorescence recovery after photobleaching

*E. coli* lipid extract was doped with 0.5 mol% Topfluor PC by mixing the respective lipids in chloroform and preparing SUVs^27^. A thin mica slide was suspended over a 10 mm glass window in a glass bottomed cell culture plate. Deposition of fluorescently doped SUVs was achieved as described above, taking care to ensure that the mica support remained hydrated at all time. Lipid bilayers were transferred to a FV1200 confocal microscope equipped with a 100 × 1.40 oil immersion objective (both Olympus) and a TC-324B automatic temperature controller (Warner Instruments) set to 37 °C. The microscope was further set to a FV10-LD473 473 nm and 15 mW laser diode powered by a FV10-MCPSU power supply, and the Alexa 488 excitation filter and BA490–590 emission filter sets (all Olympus). The fluorescent bilayers were imaged at 2.5% laser output power across 49.8 µm wide areas and an acquisition speed of 1.64 s per image. FRAP was performed on a circular area of 16.4 µm diameter at the centre of an image for 2 s at a laser output of 80%. Analysis of the FRAP data was performed according to the Soumpasis model for diffusion limited recovery^24,42^.

### Kinetic analysis

Given that the initiation and prolongation of C9 binding to C5b-8 occur at such different time scales (*τ*_init_ and *τ*_olig_ or *τ*_+_, respectively), we approximate the reaction kinetics by considering the separate reactions:

2 $${\mathrm{C5b}}{\hbox{-}}8 + {\mathrm{C}}9\mathop { \to }\limits^{k_{{\mathrm{init}}}} {\mathrm{C5b}}{\hbox{-}}8{\mathrm{(C9)}}_1$$

and

3 $${\mathrm{C5b}}{\hbox{-}}8{\mathrm{(C9)}}_n + {\mathrm{C}}9\mathop { \to }\limits^{k_ + } {\mathrm{C5b}}{\hbox{-}}8{\mathrm{(C9)}}_{n + 1}\left( {1 \le n \ < \ 18} \right),$$

while assuming an excess (and therefore approximately constant) amount of C9 in solution (see main text).

The kinetics of the initiation reaction then follows from the differential equation

4 $${\mathrm{d}}\left[ {{\mathrm{C5b}}}{\hbox{-}}8{\mathrm{(C9)}}_{\mathrm{n}} \right]{\mathrm{/d}}t = k_{{\mathrm{init}}}\left[ {{\mathrm{C5b}}}{\hbox{-}}8 \right]\left[ {{\mathrm{C9}}} \right],$$

where square brackets denote concentrations. For a single C5b-8 complex, we define the initiation probability:

5 $$p_{{\mathrm{init}}} \equiv \left[ {{\mathrm{C5b}}}{\hbox{-}}8{\mathrm{(C9)}}_1 \right]/\left( {\left[ {{\mathrm{C5b}}}{\hbox{-}}8 \right] + \left[ {{\mathrm{C5b}}}{\hbox{-}}8{\mathrm{(C9)}}_1 \right]} \right).$$

This leads to a solution of the form:

6 $$p_{{\mathrm{init}}} = 1 - {\mathrm{exp}}\left( { - k_{{\mathrm{init}}}\left[ {{\mathrm{C9}}} \right]t} \right).$$

such that *k*_init_≈*τ*_init_^−1^[C9]^−1^, with *τ*_init_ determined as illustrated in Fig. 3b.

Defining

7 $$p_n \equiv [{\mathrm{C5b}}{\hbox{-}}8{\mathrm{(C9)}}_n]/\mathop {\sum}\nolimits_{n = 1}^{18} {[{\mathrm{C5b}}{\hbox{-}}8 {\mathrm{(C9)}}_n} ],$$

we can describe the subsequent C9 oligomerization via the coupled differential equations

8 $${\mathrm{d}}p_1{\mathrm{/d}}t = - k_ + \left[ {{\mathrm{C9}}} \right]p_1$$

and

9 $${\mathrm{d}}p_n{\mathrm{/d}}t = k_ + \left[ {{\mathrm{C9}}} \right]p_{n - 1} - k_ + [{\mathrm{C9}}]p_n$$

for 2 ≤ *n* < 18, and d*p*_18_/d*t* = *k*_+_[C9]*p*_17_.

The average oligomerization rate is given in number of added C9 molecules per unit of time, as

10 $${\mathrm{d}}\left\langle n \right\rangle {\mathrm{/d}}t = \mathop {\sum}\nolimits_{n = 1}^{18} {n\,{\mathrm{d}}p_n{\mathrm{/d}}t = k_ + } \left[ {{\mathrm{C9}}} \right]\left( {\mathop {\sum}\nolimits_{n = 2}^{18} {n\,p_{n - 1}} - \mathop {\sum}\nolimits_{n = 1}^{17} {n\,p_n} } \right) \\ = k_ + \left[ {{\mathrm{C9}}} \right]\left( {\mathop {\sum}\nolimits_{n = 1}^{17} {(n + 1)p_n} - \mathop {\sum}\nolimits_{n = 1}^{17} {n\,p_n} } \right) = k_ + \left[ {{\mathrm{C9}}} \right]\mathop {\sum}\nolimits_{n = 1}^{17} {p_n} .$$

Taking into account that $\mathop {\sum}\nolimits_{n = 1}^{18} {p_n = 1}$ and assuming that *p*_18_ is significantly smaller than 1 (i.e., ignoring the oligomerization of the last few C9s), we find a constant oligomerization rate

11 $${\mathrm{d}}\left\langle n \right\rangle {\mathrm{/d}}t \approx k_ + \left[ {{\mathrm{C9}}} \right],$$

such that

12 $$k_ + \approx \tau _ + ^{ - 1}\left[ {{\mathrm{C}}9} \right]^{ - 1},$$

with *τ*_+_ determined as in Fig. 4.

### Reporting summary

Further information on research design is available in the Nature Research Reporting Summary linked to this article.

## Supplementary information

Supplementary Information

Supplementary Movie 1

Supplementary Movie 2

Supplementary Movie 3

Supplementary Movie 4

Description of Additional Supplementary Files

Reporting Summary

Source Data

## Data Availability

Data supporting the findings of this manuscript are available from the corresponding authors upon reasonable request. A reporting summary for this Article is available as a Supplementary Information file. The source data underlying Figs. 2b, 3b, 4b,c and Supplementary Figs. 2c, 3c–e, 7a–b, 8a–b and 9b are provided as a Source Data file.

## Code availability

Analysis code to extract kinetic parameters from rapid AFM imaging data is available on github. An example AFM data set is provided as a MATLAB data structure to enable assessment of the analysis code.
