# Supplementary material for: Single-molecule kinetics of pore assembly by the membrane attack complex
Source: Nat Commun. 2019 May 6;10:2066. doi: 10.1038/s41467-019-10058-7 (PMC6502846; doi:10.1038/s41467-019-10058-7)
Supplement: Supplementary file 1 — Supplementary Information [file 41467_2019_10058_MOESM1_ESM.pdf]

Single-molecule kinetics of pore assembly by the membrane attack complex

Edward S. Parsons, George J. Stanley, Alice L. B. Pyne, Adrian W. Hodel, Adrian P. Nievergelt, Anaïs Menny, Alexander R. Yon, Ashlea Rowley, Ralf P. Richter, Georg E. Fantner, Doryen Bubeck, and Bart W. Hoogenboom

Supplementary Information

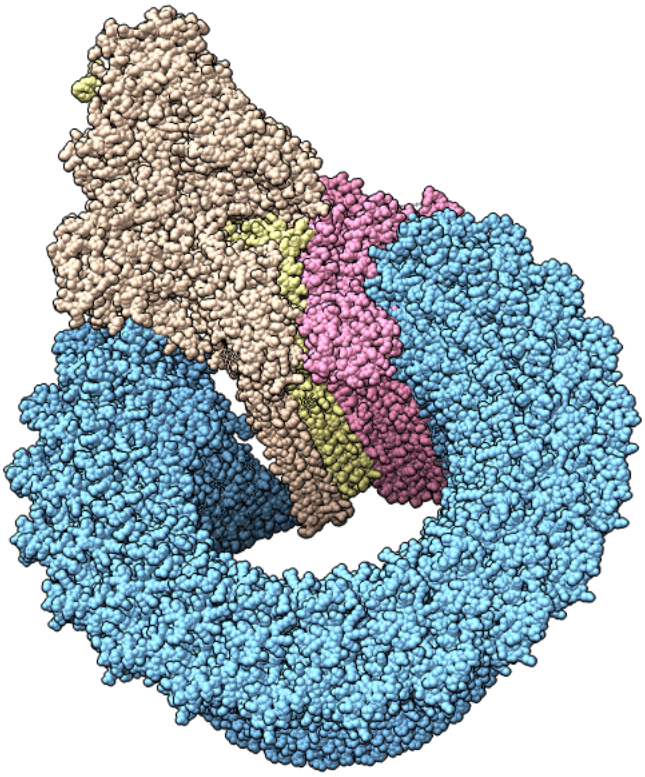

Supplementary Figure 1. The structure of the membrane attack complex. Tan: C5b6. Yellow: C7. Pink: C8. Blue: C9. Rendered from PDB 6H04[1].

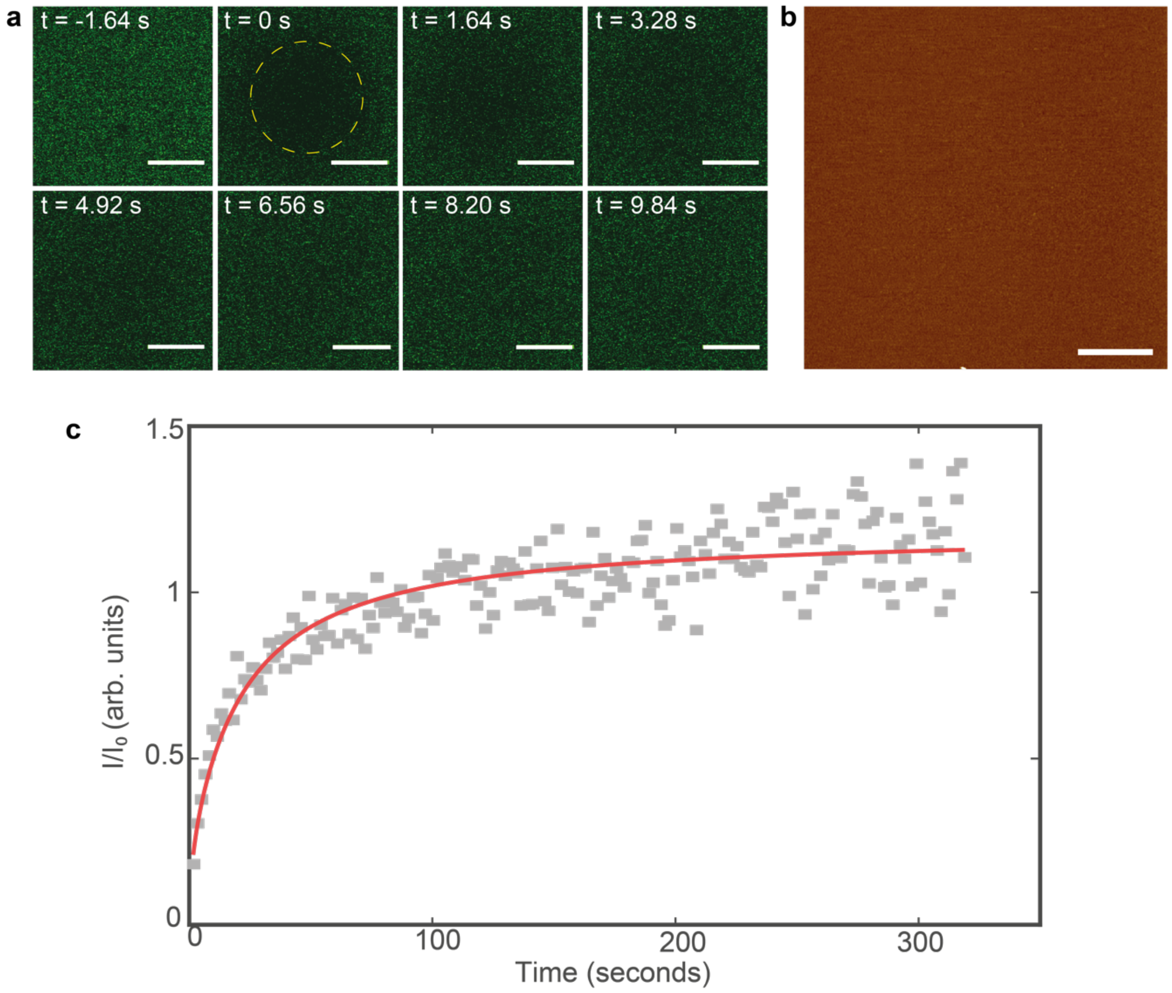

Supplementary Figure 2. Fluidity of bacterial model membranes. a, Fluorescence recovery after photobeaching (FRAP) of a supported lipid bilayer of *E. coli* lipid extract, doped with 0.5 mol % TopFluor-PC, on mica. The FRAP signal demonstrates the rapid diffusion of lipid molecules within the plane of the bilayer. Scale bars: 5 μm. Region highlighted by yellow dashed circle shows region of bleaching. **b**, AFM data showing a supported lipid bilayer of *E. coli* lipid extract on mica. Scale bar: 400 nm. Height scale as in Fig. 1a. **c**, Double-normalized (see Methods) fluorescence recovery curve for the data set shown in **a**. The diffusion coefficient for lipids within the supported bilayer was extracted from the data in **a** according to the Soumpasis model for diffusion limited recovery[2] (fit shown by red line), and calculated to be 2.26 ± s.d. 0.38 $\mu m^2\ s^{-1}$ (data taken from 5 bleached areas across 2 supported bilayer samples).

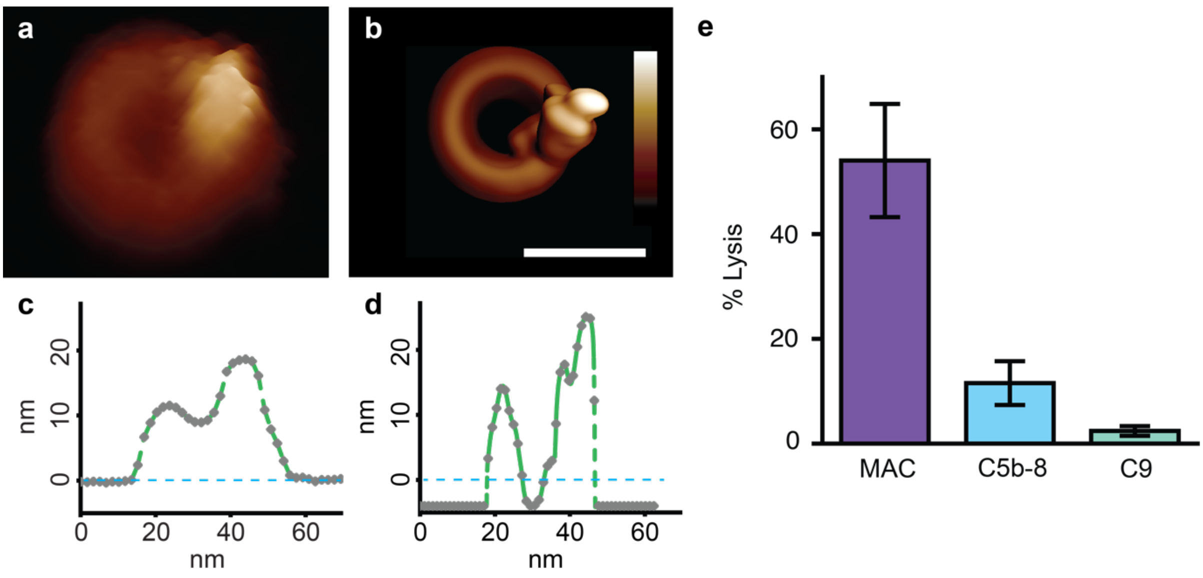

Supplementary Figure 3. Comparison of AFM data with cryo-electron microscopy reconstruction, and functional lysis assay. a, AFM image of a MAC pore. **b**, Cryo electron microscopy reconstruction of a MAC pore (EMD-3134) and rendered in Chimera with a Gaussian filter with $\sigma = 14$ pixels (with 1 pixel corresponding to the voxel size of 0.28 nm). **c**, AFM height profile taken diagonally across the MAC pore and including the C5b6 stalk, with the height of the membrane defined as 0 nm and marked by a blue dashed line. The stalk extends to 18.5 nm above the membrane whilst the C9 oligomeric ring sits at approximately 11.5 nm. The pore lumen is observed as a minimum between the stalk and the C9 rim. **d**, Height profile taken horizontally across the EM data set including the stalk. The virtual height of the membrane is defined as 0 nm using a 4 nm offset (taken as the approximate width of a lipid bilayer) with respect to the bottom of the pore, and marked by a blue dashed line. **e**, Vesicle lysis assay showing the requirement of C5b-8 + C9 (MAC) for efficient lysis of an *E. coli* membrane mimic, as determined from calcein release upon rupture of loaded small unilamellar vesicles. Scale bar a,b**:** 20 nm. Height scale (inset in b) a, b: 30 nm.

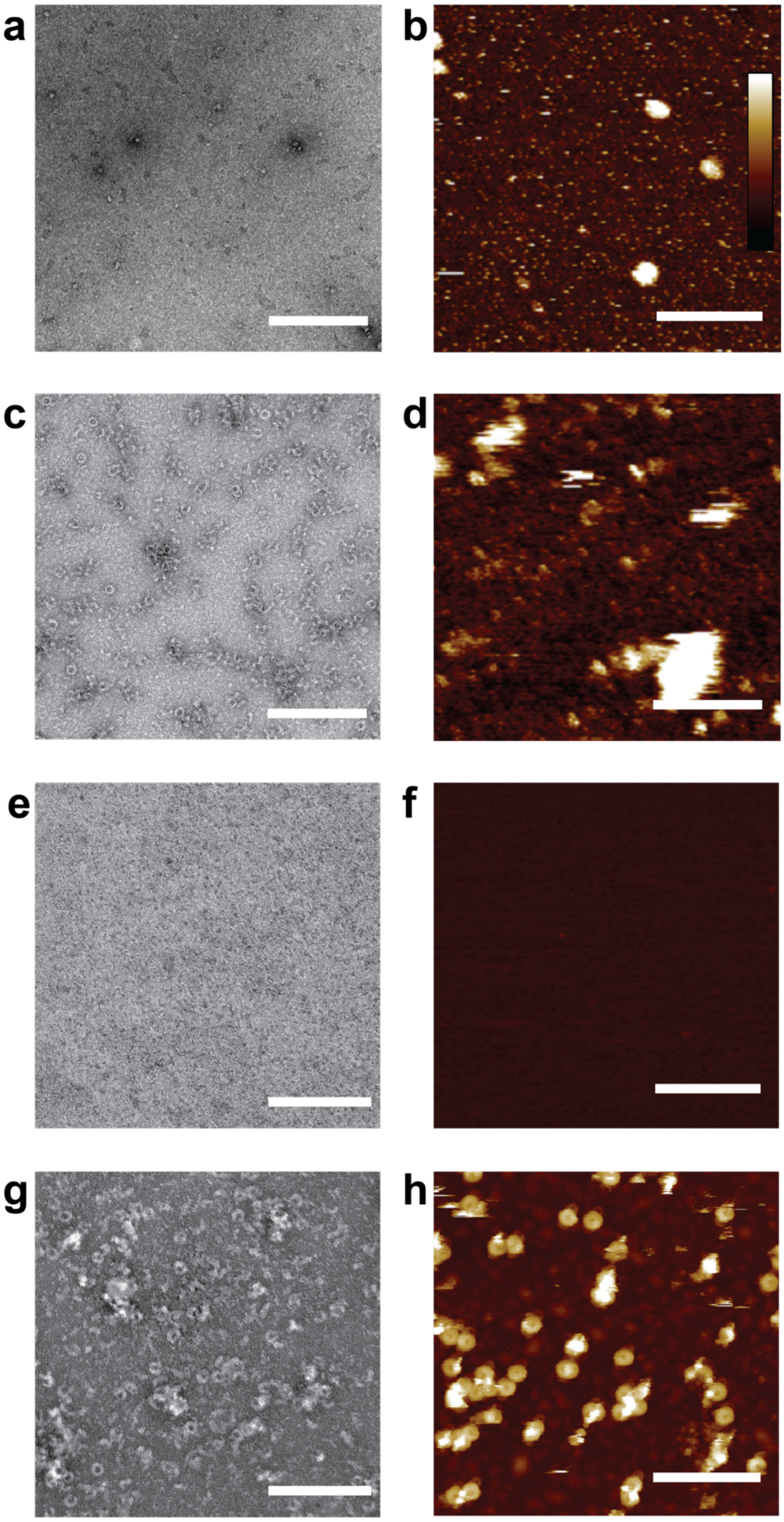

Supplementary Figure 4. Negative stain electron microscopy (a, b, c, d) and AFM (e, f, g, h) controls. a, b, C9 deposited on a (a) glow-discharged carbon grid or a (b) bare mica surface at room temperature, showing no C9 oligomerization in the absence of lipids, as no

poly-C9 rings are observed. **c**, **d,** C9 deposited on a (c) glow-discharged carbon grid or a (d) bare mica surface at 37 ºC. At 37 ºC, C9 shows some oligomerization in solution. EM shows such short C9 oligomers and complete polymerized C9 (poly-C9) rings on the grid. These oligomers appear in multiple orientations on the grid, indicating the absence of a membrane which, if present, would align the complex with the pore lumen perpendicular to the EM grid. AFM does not appear to resolve these states and instead shows aggregates of protein on the mica surface. **e, f,** A supported bilayer of *E. coli* lipid extract formed (e) on a silicon dioxide grid or (f) on a mica surface, incubated with C9 at 37 ºC, showing no C9 assemblies on the membrane by either technique, under the conditions used in our experiments (hence the poly-C9 shown in c,d does not bind to the membrane). **g, h,** A supported bilayer of *E. coli* lipid extract formed on a (g) silicon dioxide grid or (h) thermal silicon oxide surface sequentially incubated with C5b6, C7, C8 and C9 at 37 ºC. We may attribute the lower height objects (light brown) in h to the inherent surface roughness of the silicon dioxide surface, which defines the contours of the deposited bilayer. Complete oligomeric MAC pores are visible in both images, with the pores aligned perpendicular to the membrane. The surface density of MAC pores in (g) and (h) is ca. 120 pores/μm^2, showing consistent results between both methodologies (i.e. supported bilayers on mica as imaged by AFM versus negatively-stained supported lipid bilayers on silicon dioxide as imaged by TEM. Scale bars: 200 nm. Height scale (inset in b): 20 nm (b,d,f), 30 nm (h).

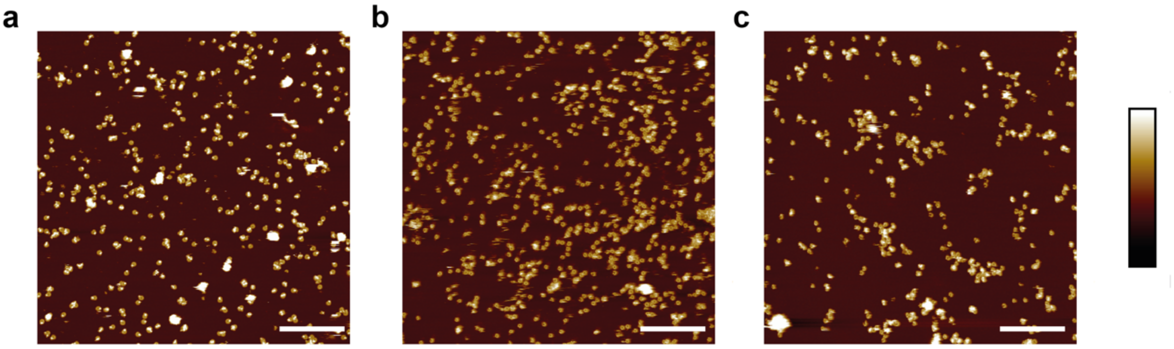

Supplementary Figure 5. AFM data showing equivalence of MAC pores formed in lipid bilayer membranes of the different compositions used in this study. a, *E. coli* total lipid extract. **b,** DOPE:DOPG (50:50 mol %). **c,** DOPC:DOPE:DOPG (47.5:47.5:5 mol %). Scale bar: 400 nm. Height scale: 30 nm.

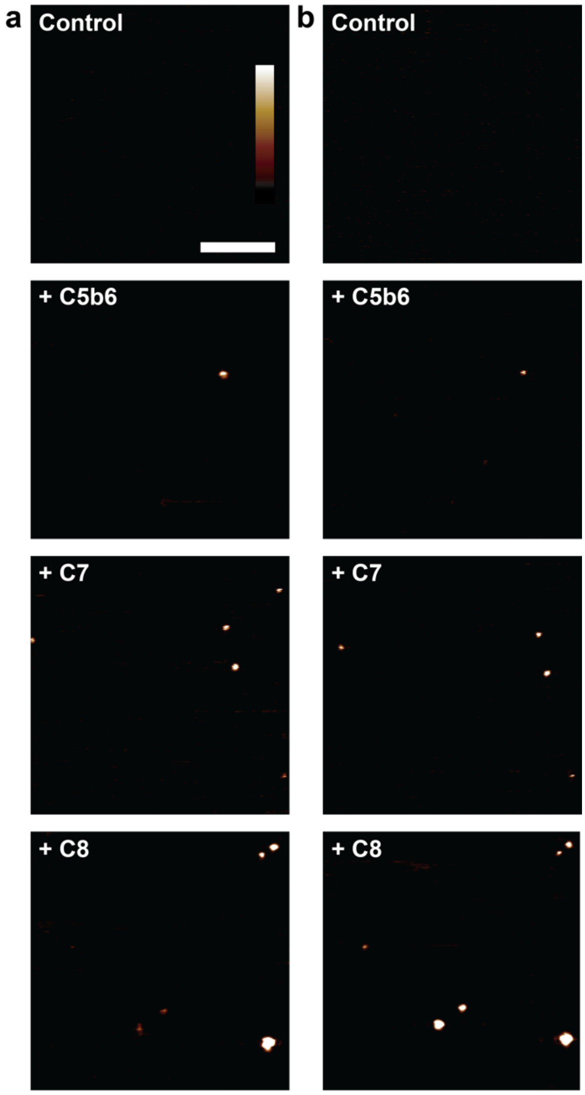

Supplementary Figure 6. Observation of MAC precursors by AFM. AFM images from the same data set shown in Fig.2a, demonstrating the stable nature of complement proteins C5b6, C7 and C8 on the supported bilayer (DOPE:DOPG, 50:50 mol %). Bacterial model membrane without the addition of complement proteins (control); in the presence of C5b6 only (+C5b6); in the presence of C5b6 and C7 (+C7); and in the presence of C5b6, C7 and C8 (+C8). **a** and **b** are separated by 135 seconds**.** Scale bar (inset in a): 200 nm. Height scale: (inset in a): 1 nm.

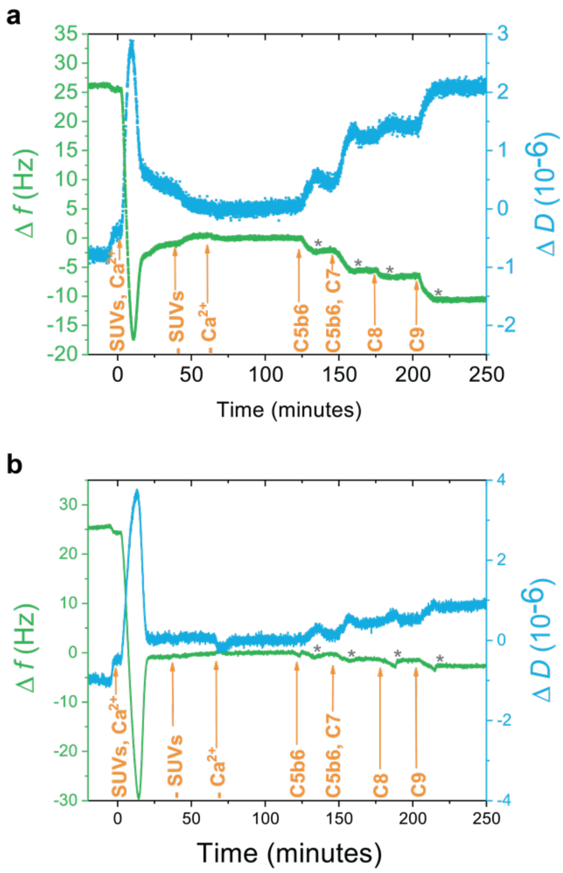

Supplementary Figure 7. QCM-D traces showing the formation of supported lipid bilayers and subsequent complement protein binding as MAC assembly proceeds. a, QCM-D shows the formation of a continuous bilayer[3] of DOPC:DOPE:DOPG (47.5:47.5:5 mol %) on a silicon dioxide substrate, and subsequent complement protein binding. Wash steps (*) followed each addition. **b**, As a, on a DOPC bilayer. Identical protein concentrations and flow rates were used for both systems. Substantial and stable binding to PG containing bilayers (a) is observed, with a weaker (and largely reversible) signal recorded for DOPC (b). For ease of comparison, all frequency (Δ*f*) and dissipation (Δ*D*) data were offset to zero at the end of the bilayer formation process.

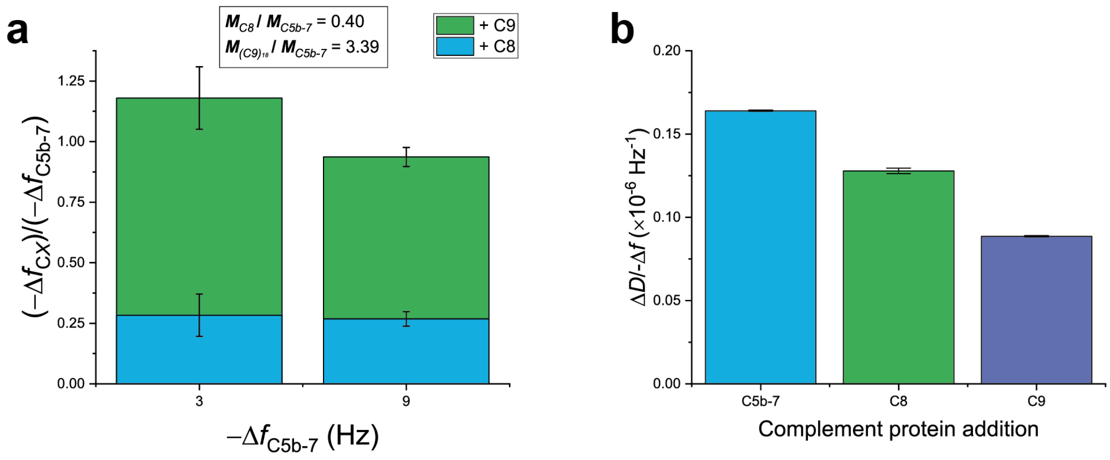

Supplementary Figure 8: QCM-D analysis of MAC stoichiometry and rigidity. a, Frequency shift upon binding of C8 and C9 ("CX", see colour coding and legend), respectively, relative to the frequency shift upon C5b-7 binding; data for two distinct C5b-7 surface coverages are shown, corresponding to frequency shifts of -3 Hz and -9 Hz, respectively. The $\Delta f_{CX}/\Delta f_{C5b\text{-}7}$ ratios are here taken as a relative measure of the CX:C5b-7 stoichiometry, and their approximate invariance to C5b-7 coverage indicates that the stoichiometry of the complex is set by the initial membrane binding of C5b-7. Note that the absolute stoichiometry cannot be quantified from QCM-D data alone because the amounts of hydrodynamically coupled solvent (which contribute along with the biomolecular masses to the frequency shifts[4]) depend on the protein structure and organization on/in the membrane. They are unknown for the system considered here; to illustrate this, the inset shows the expected mass ratios ($M_{CX}/M_{C5b\text{-}7}$) for the MAC, which are very different from the $\Delta f_{CX}/\Delta f_{C5b\text{-}7}$ ratios. **b,** ΔD/-Δf ratios for the binding of C5b-7, C8 and C9, respectively. The ΔD/-Δf ratio reflects the compliance (or 'softness') of the surface adlayer and/or its constituents[4]. The drop in ΔD/-Δf here reflects the more rigid appearance of the membrane-inserting C8 and C9 as compared to the membrane-bound C5b-7. Standard errors are extracted from the linear fit to ΔD/-Δf vs. -Δf for each binding event respectively.

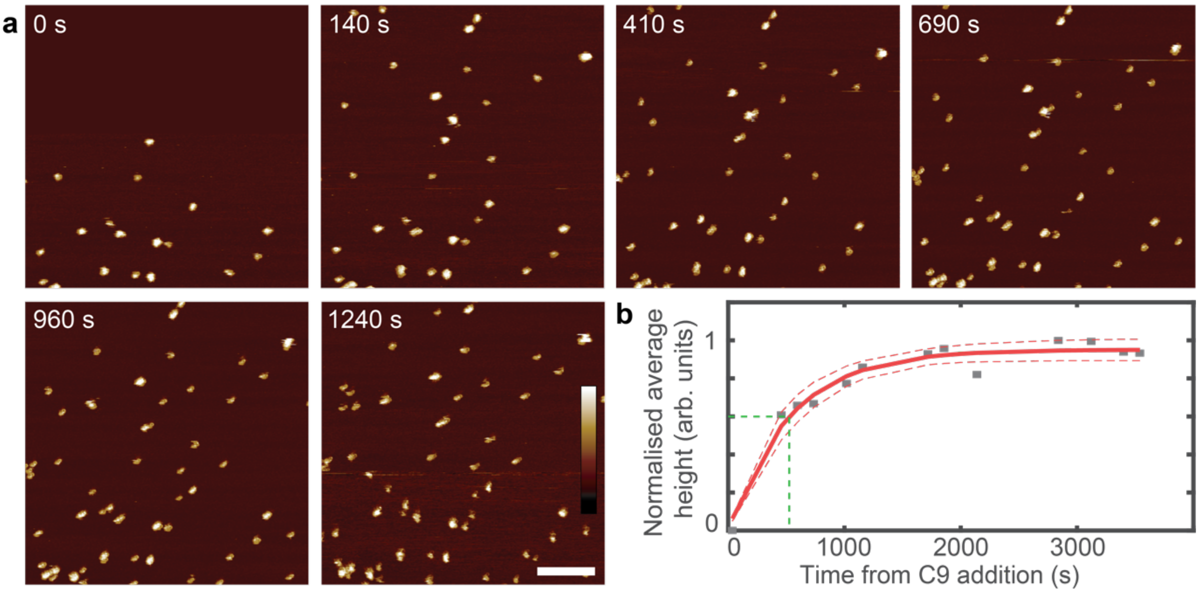

Supplementary Figure 9. Time-lapse AFM imaging of MAC assembly on supported bilayers formed of *E. coli* lipid extract at 37 °C. a, Sequence of AFM images after the addition of C9, recorded at 37 °C (see Supplementary Video 2 for full data set). For the images shown in **a**, C9 was added immediately before recording the first frame, which is scanned from top to bottom by the AFM probe. Probably due to the presence of poly-C9 oligomers in solution under these conditions (as shown in Supplementary Fig. 2), image resolution tends to be poorer than for data recorded at lower temperatures, as debris is picked up by the scanning AFM probe. Scale bar: 400 nm; height scale: 30 nm. **b,** Quantification of MAC pores on the membrane from Supplementary Video 2, via the mean height of each frame; green squares represent normalised height (see Methods). The solid red line represents fitting with the function $A\,(1 - \exp(-t/\tau_{\text{init}}))$, where $\tau_{\text{init}} = 530 \pm 109\ s$ represents the rate of MAC pore appearance (approximately 2× faster than is the case at 30 °C, Fig. 3). Green dashed lines highlight τ_{init}. Dotted red lines indicate the 95% confidence interval of the fit.

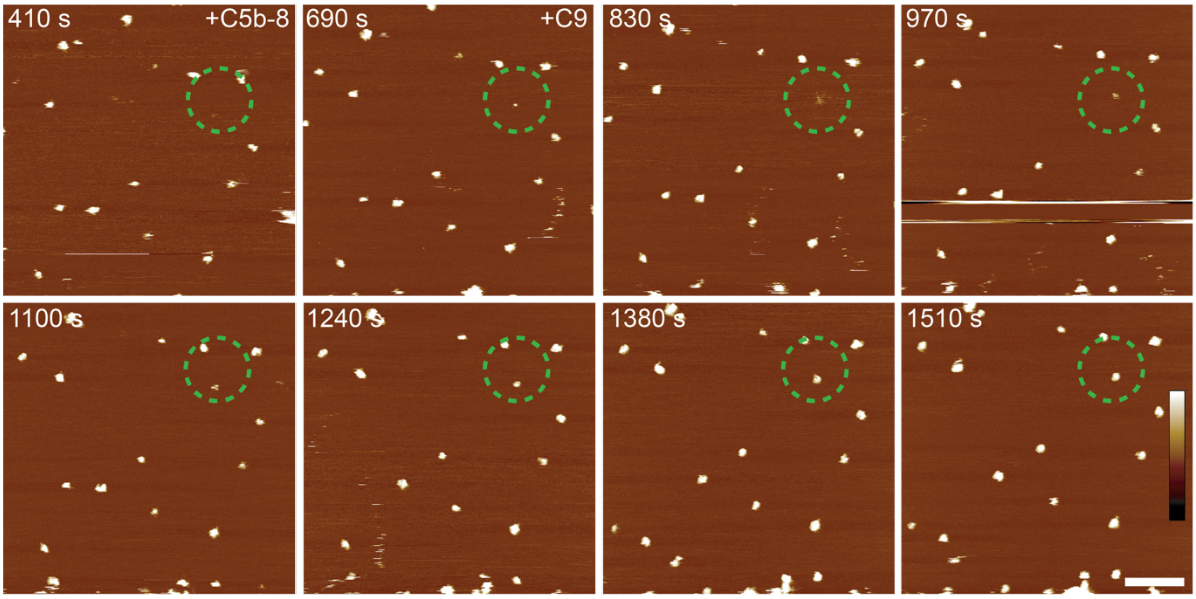

Supplementary Figure 10. Time-lapse AFM imaging of MAC assembly on supported bilayers formed of *E. coli* lipid extract, with increased duration of C8 incubation. After 1 hour of incubation with C5b6, C7 and C8 at 37 °C on an *E. coli* lipid bilayer, multiple aggregates are observed; i.e., prolonged incubation of C5b-8 does not facilitate more efficient MAC assembly under these conditions. In spite of this C5b-8 aggregation, upon subsequent addition of C9 (t = 690 s) at room temperature, there are assembly events that occur at the same time scale as the C9 oligomerization events in Fig. 4. An example of an assembling MAC pore is highlighted by dotted green circle, in which a MAC pore is observed to form 550 seconds after the addition of C9. Scale bar 300 nm; height scale: 30 nm. For full data set, see Supplementary Video 3.

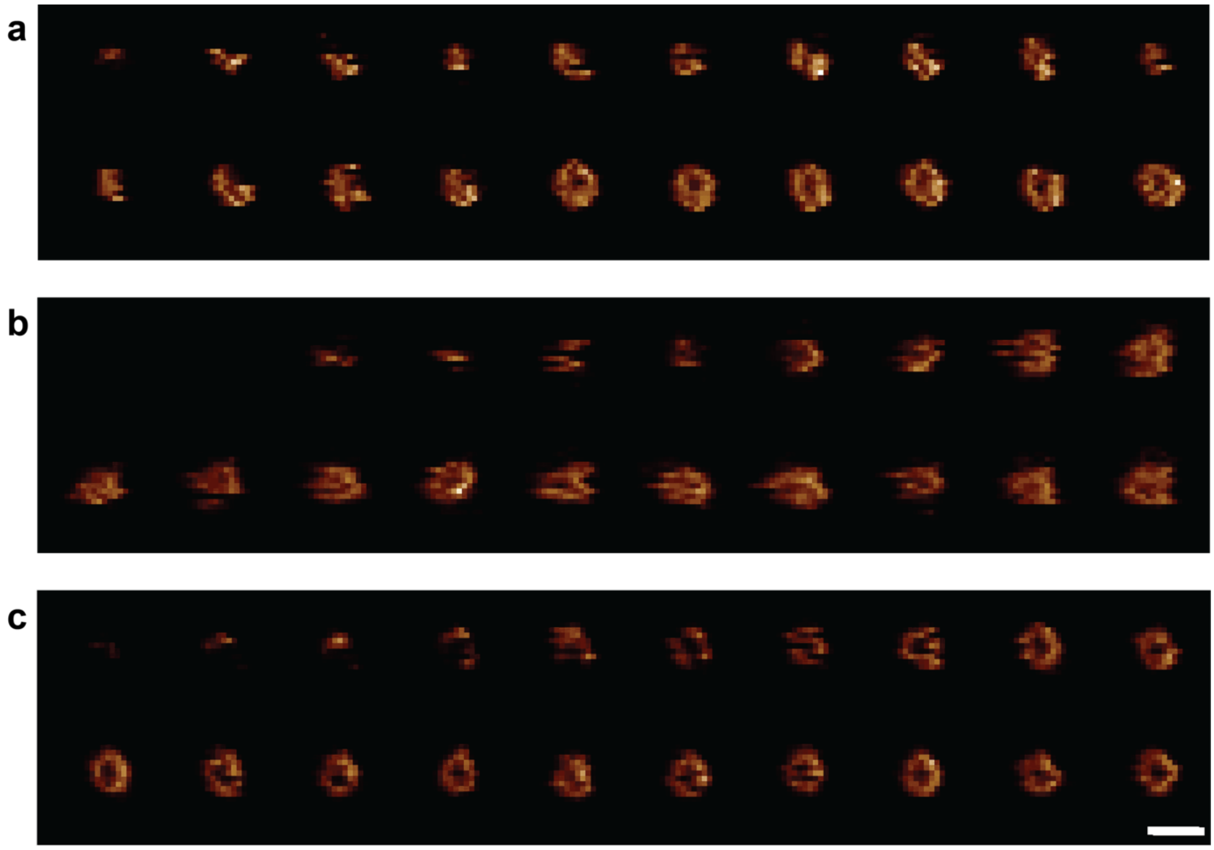

Supplementary Figure 11. Real-time visualization of C9 oligomerization. AFM sequences (from top left to bottom right) of MAC completion by C9 oligomerization for three separate pore formation events recorded at 6.5 seconds per frame, extracted from the data set presented in Fig. 3 and Supplementary Video 1. Scale bar: 30 nm, height scale (colour scale, see Supplementary Figs. 2-4, 6, 7): 16 nm.

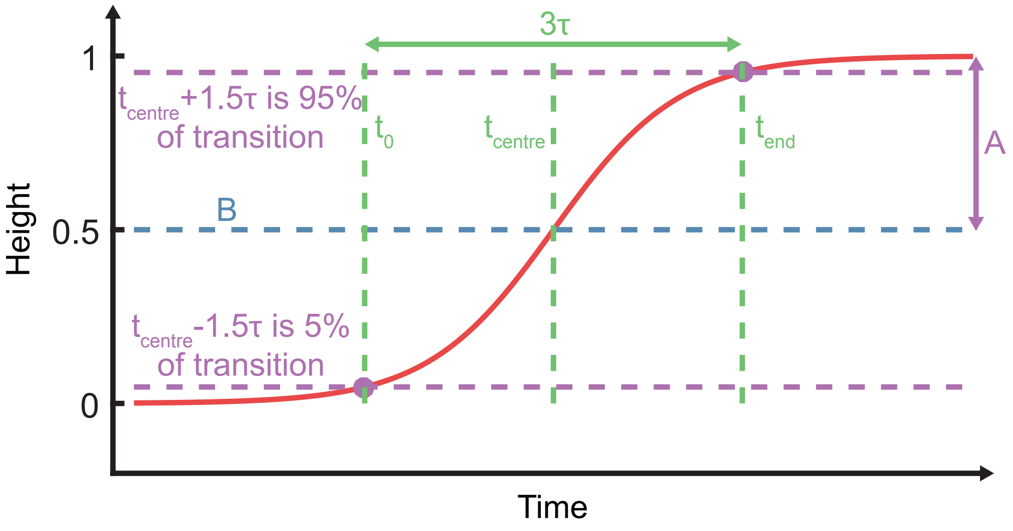

Supplementary Figure 12. Estimation of the C9 oligomerization time. The oligomerization time for individual pores is estimated by fitting data as in Fig. 4 with a sigmoidal function $A * \tanh\left(\frac{t-t_{\mathrm{centre}}}{\tau}\right) + B$, as a generic and mathematically convenient model for a smooth transition between pore absence and pore completion. A is the amplitude of the sigmoidal fit, t is time (seconds), t_{centre} is the time at the centre of the transition, and τ is ~46% of the width of the transition. The oligomerization time τ_{olig} is determined from the width of the transition, which in this form is taken as the time needed to cover 90% of the transition, $\tau_{\mathrm{olig}} = 3\tau$.

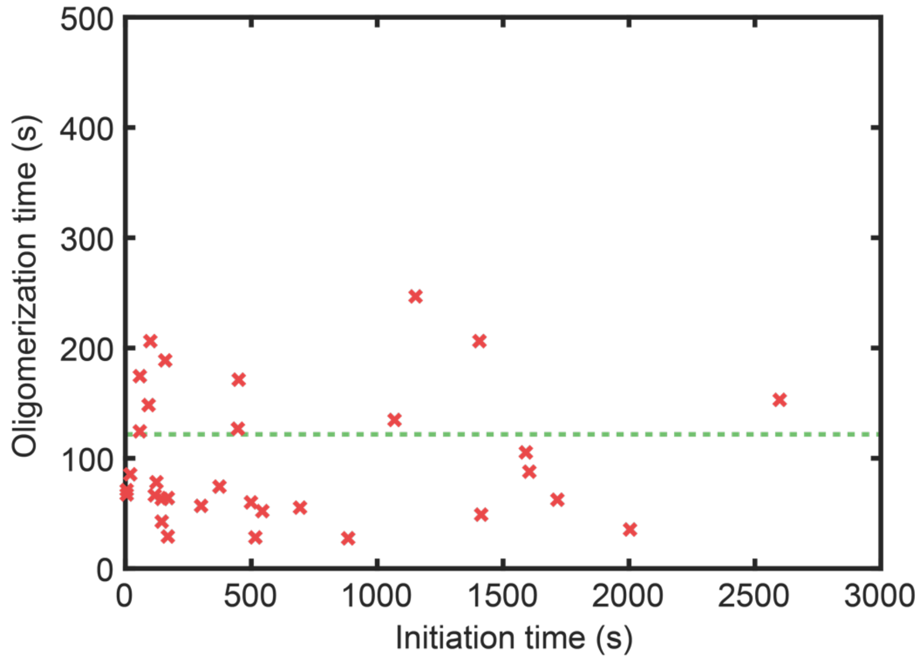

Supplementary Figure 13. Oligomerization time is independent of initiation time. C9 oligomerization time for individual MAC pores, as a function of the time at which each pore first appears in the images (initiation time, referred to time of C9 injection), based on data as shown in Fig. 4. Dotted green line represents the mean oligomerization time (112 s), as calculated from Fig 4c.

Supplementary References

1. Menny, A. *et al.* CryoEM reveals how the complement membrane attack complex ruptures lipid bilayers. *Nat Commun* **9,** 5316 (2018).
2. Soumpasis, D. M. Theoretical analysis of fluorescence photobleaching recovery experiments. *Biophys. J.* **41,** 95–97 (1983).
3. Richter, R. P., Bérat, R. & Brisson, A. R. Formation of solid-supported lipid bilayers: an integrated view. *Langmuir* **22,** 3497–3505 (2006).
4. Reviakine, I., Johannsmann, D. & Richter, R. P. Hearing what you cannot see and visualizing what you hear: Interpreting quartz crystal microbalance data from solvated interfaces. *Anal. Chem.* **83,** 8838–8848 (2011).
