# Supplementary material for: Single-molecule kinetics of pore assembly by the membrane attack complex
Source: Nat Commun. 2019 May 6;10:2066. doi: 10.1038/s41467-019-10058-7 (PMC6502846; doi:10.1038/s41467-019-10058-7)
Supplement: Supplementary file 6 — Description of Additional Supplementary Files [file 41467_2019_10058_MOESM6_ESM.docx]

**Description of Additional Supplementary Files**

**Title: Supplementary Video 1. Real-time visualization of MAC formation at 30 ^o^C.**
AFM sequence showing effect of subsequent incubations of MAC components C5b6, C7, C8 and C9 on a bacterial model membrane. For snapshots and analysis, see Fig. 3.

**Title: Supplementary Video 2. Real-time visualization of MAC formation at 37 ^o^C.**
AFM sequence showing effect of subsequent incubations of MAC components C5b6, C7, C8 and C9 on a bacterial model membrane. For snapshots and analysis, see Supplementary Fig. 6.

**Title: Supplementary Video 3. Real-time visualization of MAC formation following prolonged incubation of C5b6, C7 and C8 at 37 ^o^C.**
AFM sequence showing effect of subsequent incubations of C9 on a bacterial model membrane (at room temperature), following 1 hour incubation with C5b6, C7, C8. For snapshots and analysis, see Supplementary Fig. 7.

**Title: Supplementary Video 4. Real-time visualization of C9 oligomerization for single pore formation events.**
Average height with respect to time is plotted to show tracking of MAC pore formation.
