# Supplementary material for: Single-molecule kinetics of pore assembly by the membrane attack complex
Source: Nat Commun. 2019 May 6;10:2066. doi: 10.1038/s41467-019-10058-7 (PMC6502846; doi:10.1038/s41467-019-10058-7)
Supplement: Supplementary file 7 — Reporting Summary [file 41467_2019_10058_MOESM7_ESM.pdf]

nature research

Corresponding author(s): Edward Parsons & Bart Hoogenboom

Reporting Summary

Nature Research wishes to improve the reproducibility of the work that we publish. This form provides structure for consistency and transparency in reporting. For further information on Nature Research policies, see Authors & Referees and the Editorial Policy Checklist.

Statistical parameters

When statistical analyses are reported, confirm that the following items are present in the relevant location (e.g. figure legend, table legend, main text, or Methods section).

n/a	Confirmed	
☐	☒	The exact sample size (n) for each experimental group/condition, given as a discrete number and unit of measurement
☐	☒	An indication of whether measurements were taken from distinct samples or whether the same sample was measured repeatedly
☒	☐	The statistical test(s) used AND whether they are one- or two-sided<br>*Only common tests should be described solely by name; describe more complex techniques in the Methods section.*
☒	☐	A description of all covariates tested
☒	☐	A description of any assumptions or corrections, such as tests of normality and adjustment for multiple comparisons
☐	☒	A full description of the statistics including central tendency (e.g. means) or other basic estimates (e.g. regression coefficient) AND variation (e.g. standard deviation) or associated estimates of uncertainty (e.g. confidence intervals)
☒	☐	For null hypothesis testing, the test statistic (e.g. F, t, r) with confidence intervals, effect sizes, degrees of freedom and P value noted<br>*Give P values as exact values whenever suitable.*
☒	☐	For Bayesian analysis, information on the choice of priors and Markov chain Monte Carlo settings
☒	☐	For hierarchical and complex designs, identification of the appropriate level for tests and full reporting of outcomes
☒	☐	Estimates of effect sizes (e.g. Cohen's d, Pearson's r), indicating how they were calculated
☐	☒	Clearly defined error bars<br>*State explicitly what error bars represent (e.g. SD, SE, CI)*

Our web collection on statistics for biologists may be useful.

Software and code

Policy information about availability of computer code

Data collection	Bruker's Nanoscope v. 8 was used in the collection of AFM data. Biolin Scientific's 'QTools' was used in the collection of QCM-D data. FRAP data was collected using Olympus's Fluoview v. 4.2b. NS-EM data was collected with FEI Tecnai Microscope control, and Gatan Digital Micrograph software controlling the camera.
Data analysis	Analysis of AFM data was performed using Bruker's Nanoscope analysis v. 1.8. Custom analysis of AFM movies was performed using the code provided (MATLAB, MathWorks). Analysis of QCM-D data was performed using Biolin Scientific's 'QSoft'. Analysis of FRAP image sequences and NS-EM was performed using FIJI (is just imageJ).

For manuscripts utilizing custom algorithms or software that are central to the research but not yet described in published literature, software must be made available to editors/reviewers upon request. We strongly encourage code deposition in a community repository (e.g. GitHub). See the Nature Research guidelines for submitting code & software for further information.

Data

Policy information about availability of data

All manuscripts must include a data availability statement. This statement should provide the following information, where applicable:

- Accession codes, unique identifiers, or web links for publicly available datasets
- A list of figures that have associated raw data
- A description of any restrictions on data availability

The datasets generated during the current study are available from the corresponding author on reasonable request. An example AFM dataset is provided as a MATLAB data structure to enable assessment of the analysis code.

Field-specific reporting

Please select the best fit for your research. If you are not sure, read the appropriate sections before making your selection.

☒ Life sciences ☐ Behavioural & social sciences ☐ Ecological, evolutionary & environmental sciences

For a reference copy of the document with all sections, see nature.com/authors/policies/ReportingSummary-flat.pdf

Life sciences study design

All studies must disclose on these points even when the disclosure is negative.

Sample size	The sample size for a given experiment is the number of MAC pores observed within the field-of-view during imaging. This is determined by the tracking analysis which searches for MAC pores within each image (this is done using a 2D cross-correlation routine).
Data exclusions	After running the first two tracking scripts ("MACanalysis_loadfiles_findtracks.m" and "MACanalysis_loadtracks_createTrackVideos.m"), the found tracks are reviewed manually. Any false-positive tracks, inaccurate tracks, or tracks of complete MAC pores drifting into the field-of-view are discounted. This leaves tracks of unique MAC pores oligomerising within the field-of-view.
Replication	Rapid AFM imaging experiments were repeated > 50 times on 4 unique microscopes (2 commercial and 2 custom), with consistent results at differing temporal resolutions. For the AFM data recording at 6.5 seconds per frame, 6 unique datasets were recorded, from which 33 unique oligomerization events were extracted (N.B. - an oligomerization time could not be extracted for every event counted as a pore (when determining initiation time, as in Fig. 3) owing to the data exclusions listed above).
Randomization	N/A
Blinding	N/A

Reporting for specific materials, systems and methods

Materials & experimental systems

n/a	Involved in the study
☐	☒ Unique biological materials
☒	☐ Antibodies
☒	☐ Eukaryotic cell lines
☒	☐ Palaeontology
☒	☐ Animals and other organisms
☒	☐ Human research participants

Methods

n/a	Involved in the study
☒	☐ ChIP-seq
☒	☐ Flow cytometry
☒	☐ MRI-based neuroimaging

Unique biological materials

Policy information about availability of materials

Obtaining unique materials	All purified complement proteins are available to buy from Complement Technologies (Texas, USA).

April 2018
